# ﻿Reassessment of *Leucothyreus noctivagus* Ohaus, 1917 reveals *Mimogeniates margaridae* Martínez, 1964 as new generic and specific synonym (Scarabaeidae, Rutelinae, Geniatini)

**DOI:** 10.3897/zookeys.1264.153948

**Published:** 2025-12-11

**Authors:** Matheus Bento, Paschoal Grossi, Claudio Ruy Vasconcelos da Fonseca, Matthias Seidel

**Affiliations:** 1 Instituto Nacional de Pesquisas da Amazônia (INPA), Coordenação de Biodiversidade, Laboratório de Sistemática e Ecologia de Coleoptera (LASEC), Manaus, Brazil Instituto Nacional de Pesquisas da Amazônia (INPA), Coordenação de Biodiversidade Manaus Brazil; 2 Universidade Federal Rural de Pernambuco (UFRPE), PPG em Entomologia, Laboratório de Taxonomia de Insetos (LabTaxIn), Rua Dom Manoel de Medeiros, s/n, 52171-900, Recife, Pernambuco, Brazil Universidade Federal Rural de Pernambuco (UFRPE) Recife Brazil; 3 Naturhistorisches Museum Wien, Zweite Zoologische Abteilung, Burgring 7, 1010 Vienna, Austria Naturhistorisches Museum Wien Vienna Austria

**Keywords:** Bearded scarab beetles, monotypic, morphology, Neotropical region, nomenclatural changes, taxonomy

## Abstract

In this paper, *Mimogeniates margaridae* Martínez (Scarabaeidae, Rutelinae, Geniatini) is synonymized with *Leucothyreus noctivagus* Ohaus (**syn. nov.**). Hence, the monotypic genus *Mimogeniates* Martínez is a new junior synonym of *Leucothyreus* MacLeay (**syn. nov.**). *Leucothyreus noctivagus* is redescribed and illustrated based on the type and additional material, including new distributional records in southern Brazil. As a result of our work, the tribe Geniatini now includes 12 genera.

## ﻿Introduction

The bearded scarab tribe Geniatini (Scarabaeidae, Rutelinae) is a diverse group of phytophagous scarabs widely distributed in the Neotropical Region (Jameson and Hawkins 2005). The tribe is currently composed of 338 species in 13 genera (Jameson and Hawkins 2005; Jameson 2008; Grossi and Vaz-de-Mello 2018; Ferreira et al. 2019, 2024). Members of Geniatini are diagnosed by having the labrum vertically positioned in relation to the clypeus, both the labrum and the prementum usually with a medioapical projection, the elytral margins with or without a membranous border, the inner protibial spur present, and the male protarsomeres usually enlarged and densely setose internally (Villatoro and Jameson 2001; Jameson and Hawkins 2005).

*Mimogeniates* Martínez, 1964 is a monotypic genus and was described based on *M. margaridae* Martínez, 1964 from Espírito Santo state (Brazil). The genus was erected for *M. margaridae* based on the labium having a crenulate apical margin and lacking the apical projection, which is characteristic of most geniatine genera (Martínez 1964). Because of the general form of the maxillary galea, which is “neither enlarged nor keeled at the apex”, Martínez (1964: 2) thought the genus belonged to a group formed by *Geniates* Kirby, 1818, *Rhizogeniates* Ohaus, 1909, and *Eunanus* Ohaus, 1909. However, he classified the genus near to *Bolax* Fischer & Waldheim, 1829 and *Leucothyreus* Macleay, 1819 because these genera share laterally carinate abdominal sternites (Martínez 1964; Jameson and Hawkins 2005). Nevertheless, these character states are broadly distributed across most genera of the tribe Geniatini and uninformative for genus-level taxonomy. Moreover, based on the lack of a medioapical projection on the labrum of *Mimogeniates*, Martínez (1964: 4) alluded to the superficial similarity of his new genus with Anatistini: “This new genus, due to the characters it presents with having the labrum and mentum inerm, fits perfectly in the tribe Spodochlamiini [= Anatistini], but we do not think that it should be located in this tribe, since the shape of the maxillae, mandibles, structure of the integument, secondary sexual characters and male genitalia (phallobase and parameres), are all of the Geniatini type … . Regarding *Mimogeniates*, it is not described as Spodochlamiini [= Anatistini] or as Geniatini, but due to the analysis of the qualitative characters it presents, despite the ‘fundamental’ characters of Spodochlamiini [=Anatistini], I consider it more correct to place it in Geniatini”.

The crenulate apical margin of the labium of *M. margaridae* is a unique feature among geniatine scarabs, and a morphology-based phylogenetic analysis of all Geniatini genera (Bento 2024) recovered *M. margaridae* nested in the *Leucothyreus* clade and forming a well-supported subclade with the type species, *L. kirbyanus* MacLeay, 1819. This analysis provides strong support for the synonymization of *Mimogeniates* and *Leucothyreus*. Furthermore, after analysis of the type series of both species, we found evidence that *M. margaridae* is conspecific with *Leucothyreus noctivagus* Ohaus, 1917. Based on these results, we propose the correspondent generic and specific synonyms and redescribe *L. noctivagus*.

## ﻿Methods

Type and non-type material used in this study is deposited in the following institutions (acronym and curators in parentheses): Museum für Naturkunde der Humboldt-Universität, Berlin, Germany (**MFNB**); Naturhistorisches Museum Basel, Basel, Switzerland (**NMB**; Matthias Borer and Isabelle Zürcher); and the Coleção Entomológica do Instituto Oswaldo Cruz, Rio de Janeiro, Brazil (**CEIOC**; Márcio Felix and Claudia Leal Rodrigues); Canadian Museum of Nature, Ottawa, Canada (**CMN**; François Génier); Coleção Entomológida da Universidade Federal Rural de Pernambuco (**CERPE**; Paschoal C. Grossi).

The general morphological terms follow Beutel and Lawrence (2005), with the adoption of the term **tectum** for the distal portion of the phallobase (non-apodeme *sensu*Krell 1996). Terminology regarding orientation of legs follows Fuhrmann (2021), which determines three axes: proximal/distal; inner/outer; and anterior/posterior. Cuticular surface was considered **densely setose** when the surface is completely hidden by setae; **moderately setose** when the surface is covered with setae but not hidden; and **sparsely setose** when the surface has only few setae. Body size was measured from anterior margin of clypeus to apex of pygidium, and the body width was measured at mid-elytra. The surface was considered **densely punctate** when the distance between punctures was less than twice the puncture diameter, **moderately punctate** when the distance was 2–6 times the puncture diameter, and **sparsely punctate** when the distance was more than six puncture diameters.

A Canon EOS 750D camera provided with an MP-E 65 mm macro lens was used for specimen photographs. All produced images were processed and edited using Helicon Focus (HeliconSoft) and Photoshop (Adobe Inc.). Distributional data were based on collections and published data, including Ohaus (1917), Martínez (1964), Jameson and Hawkins (2005), and Krajcik (2007). The geographic coordinates were obtained with Google Maps and the georeferenced points were plotted on the distribution map generated with Simplemappr (Shorthouse 2010).

## ﻿Results

### ﻿ Leucothyreus noctivagus

Taxon classificationAnimaliaColeopteraScarabaeidae

Ohaus, 1917

604AF01C-3E12-5B49-B998-87C4CA9F3EDB

Leucothyreus noctivagus Ohaus, 1917: 15 [original combination]; Blackwelder 1944: 249; Machatschke 1974: 409; Jameson and Hawkins 2005: 45; Krajcik 2007: 77.
Mimogeniates margaridae Martínez, 1964: 5 [original combination]; Machatschke 1972: 359; Jameson and Hawkins 2005: 24, 53, 65, 70; Krajcik 2007: 89. Syn. nov.

#### Type material examined.

**Figure 1.**
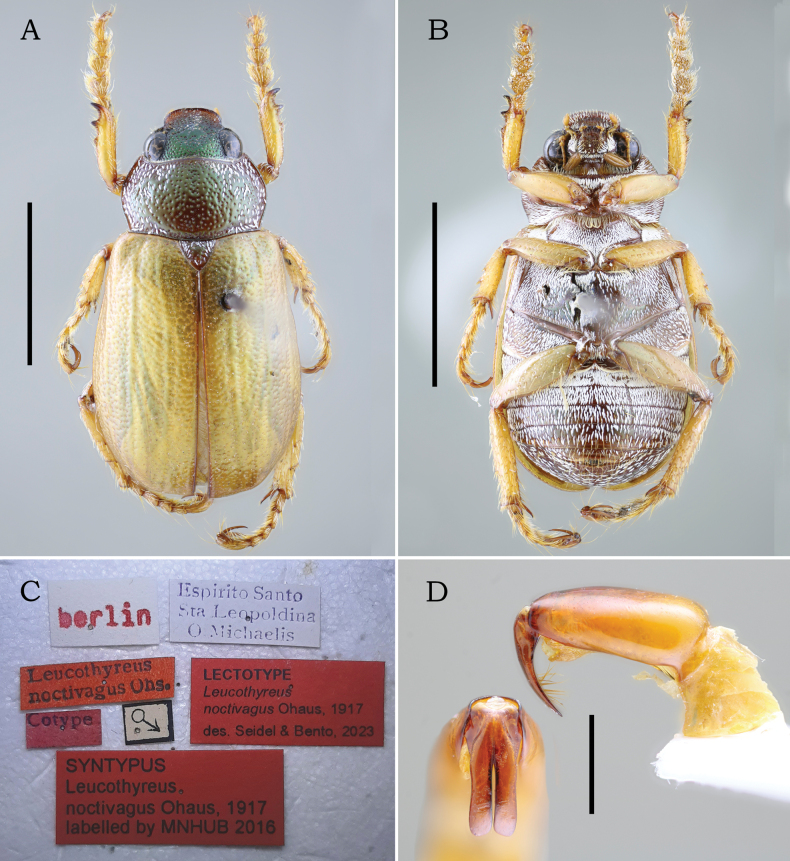
Lectotype male (here designated) of *Leucothyreus noctivagus* Ohaus, 1917. **A.** Dorsal view; **B.** Ventral view; **C.** Labels; **D.** Aedeagus in frontal and lateral views. Scale bars: 5 mm (**A, B**); 1 mm (**D**).

*Leucothyreus noctivagus*: **lectotype** • male (here designated) deposited at **MFNB** (Fig. 1A–D), labeled: “Espírito Santo / Sta. Leopoldina / O. Michaelis” (white, printed) // “berlin” (white, printed in red) // “Cotype” (red, printed) // “Leucothyreus / noctivagus Ohs.” (red, printed) // “SYNTYPUS / Leucothyreus / noctivagus Ohaus, 1817 / labeled by MNHUB 2016” (red, printed). **Paralectotypes**, • same data as lectotype (♂, **MFNB**); • same, but “Boa Sorte / F. Sahlb.” (white, printed) // “♀” (♀, **MFNB**); • same, but “Rio Itapemirim / E. Esp. S. – Brazil / 5-12-1908 / J. F. Zikán” (white, printed) (♀, **MFNB**); • same, but “Petropolis / 15.I.99 / Electr. Licht” (white, printed) (♀, **MFNB**); • same but “Minas Gerais / Mar d. Espanha / J. Zikán S.” (white, printed) // “14/11/1909” (white, handwritten) (♀, **MFNB**). *Mimogeniates margaridae*: **Paratype** • female deposited at **CMN**, labeled: “BRASIL. E. Santo / Linhares Sooretama / NOV. 62 A. Martínez” (white, printed) // “PARATIPO” (green, printed) // “Mimogeniates / margaridae / ♀ sp. n. / Martinez-det, 1964” (light green, handwritten) // “H. & A. HOWDEN / COLLECTION / ex. A. Martinez coll.” (white, printed).

#### Additional material (17 males; 17 females).

Brazil • Rio de Janeiro, Bom Jardim, Sítio São José, 29.xi.2003, M. Hoffmann col. (♂, CERPE); Brazil • São Paulo, Itú, Fazenda Pau d’Alho, 28–29.X.1965, Martins & Biasi (legs.) (♂, NMB); Brazil • Espírito Santo, Itapemirim, 5.XII.1908, J. F. Zikán (leg.) (♀, CEIOC); Brazil • Espírito Santo, Linhares, Parque Sooretama, 17–27.X.1962 (♂, CMN); Brazil • Espírito Santo, Rio Bonito, XI.1963, A. Maller (leg.), Martínez collection (♂, CMN); Brazil, Espírito Santo, Linhares, Flona de Goytacazes, 19°25'56.3"S, 40°04'12.6"W, 27.xi.2010, light, DS Martins (♂, ♀, CERPE); • same, but 09.xi.2010 (2♂, 3 ♀, CERPE); • same, but 19.xi.2010 (2 ♀, CERPE); Brazil • Bahia, Encruzilhada, XII.1980, A. Martínez & M. Alvarenga (legs.) (♀, CMN); Brazil • Minas Gerais, Pedra Azul, 1974 (♀, NMB); Brazil • Minas Gerais, Mar de Hespanha, 19.XI.1909, J. F. Zikán (leg.) (♀, CEIOC); Brazil • Minas Gerais, Ipatinga, xi.1989, luz, E. & P. Grossi legs. (5 ♂, 6 ♀, CERPE); Brazil • Minas Gerais, Itumirim, 23.3400°S, 44.6016°W, x.2016, FZ Vaz-de-Mello (♂, CERPE); Brazil • Minas Gerais, Lavras, 02.vii.2007, PF Espuri (♂, CERPE); Brazil • Minas Gerais, Berizal, Faz. Veredão, 14.xii.2007, 850 m, Grossi, Rafael & Parizotto legs. (2 ♂, ♀, CERPE); Brazil • Minas Gerais, Águas Vermelhas, 13.xii.2007, Grossi, Rafael & Parizotto legs. (♂, CERPE).

#### Diagnosis.

Labrum and prementum without medioapical projection (Fig. 3D1, D4). Apical margin of prementum crenulated (Fig. 3D4). Outer margin of metafemur coarsely crenulated, with each crenulation bearing a short, white seta (Fig. 3B). Outer margin of meso- and metatibia with two short, spine-like projections (Fig. 3A, B).

#### Differential diagnosis.

This species is similar to *L. kirbyanus* MacLeay, 1819 (see Jameson and Hawkins 2005: fig. 7), as both share a similar general appearance of head and clypeus, chaetotaxy, similar form of the male aedeagus, coarsely crenulated outer margin of metafemur (Fig. 3B) as well as the outer margin of meso- and metatibia with short, spine-like projections (Fig. 3A, B). However, *L. noctivagus* is easily distinguished from *L. kirbyanus* and other species in the genus by the form of the prementum, which is crenulated and lacking a median projection (Fig. 3A, B).

#### Description.

**Figure 2.**
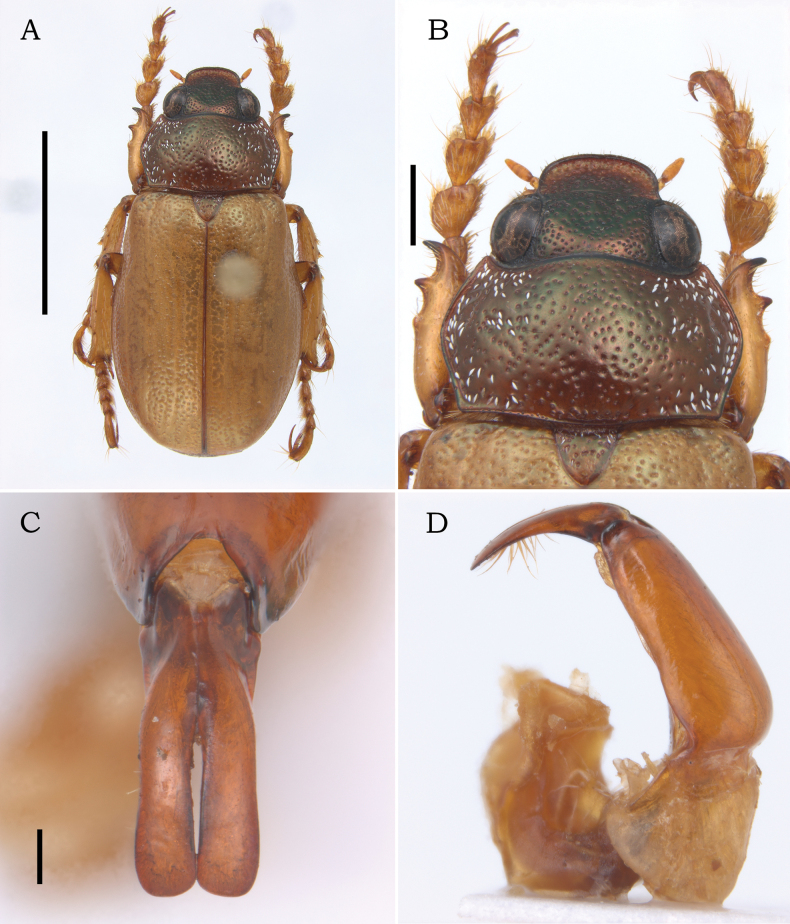
Male of *Leucothyreus noctivagus* Ohaus, 1917. **A.** Dorsal view; **B.** Head and pronotum showing color variation with weak metallic-green reflections; **C.** Parameres in frontal view; **D.** Aedeagus in lateral view. Scale bars 5 mm (**A**); 1 mm (**B**); 0.2 mm (**C**). Photos by Vitor Nardino.

**Figure 3.**
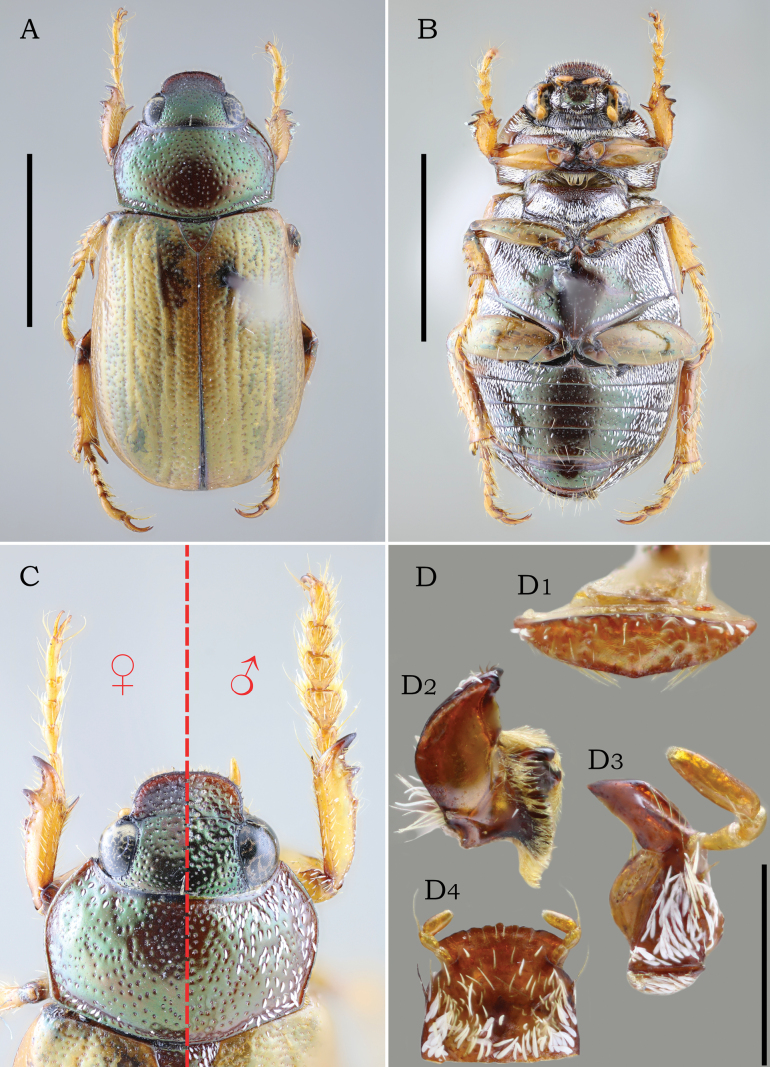
Paralectotype female of *Leucothyreus noctivagus* Ohaus, 1917. **A.** Dorsal view. **B.** Ventral view; **C.** Pronotum, head, and anterior legs of (left) female and (right) male; **D.** Mouthparts: **D1.** Labrum showing lack of apical projection; **D2.** Left mandible; **D3.** Left maxilla; **D4.** Labium showing crenulate apex. Scale bars: 5 mm (**A, B**); 1 mm (**D**).

**Male** (Figs 1A–D, 2A–D). Length 9.5–10.5 mm. Width 4.8–5.3 mm. Body oval, elongate. **Coloration** of head, pronotum, and scutellar shield reddish brown, with weak to strong metallic-green reflections (Figs 1A, 2A, B); head darker than pronotum. Elytra and legs light brown, with weak greenish reflections. Venter reddish brown, covered with white, scale-like setae. **Pubescence**: lateral surface of pronotum and scutellar shield moderately and irregularly covered with posteriorly decumbent, white, scale-like setae; setae thinner on scutellar shield; thoracic venter and abdomen densely covered with decumbent, white, scale-like setae. **Head** small, shorter than pronotum at middle, with deep, large, densely distributed punctures. Frons as wide as clypeus at base. Frontoclypeal suture complete and slightly sinuous at middle. Clypeus subrectangular, with anterior margin weakly raised. Labrum large, subtriangular, slightly longer than ventral face of clypeus; apex weakly angulated, not projected (Fig. 3D1. Labium (Fig. 3D4) subrectangular, slightly wider than long; prementum with apical margin coarsely crenulated at middle, not projected; mentum with a transverse, moderate setal brush medially erect and laterally decumbent bearing thick, white setae. Labial palp 3-segmented, with palpomere II distinctly shorter than III. Maxilla densely setose, with thick, decumbent, white setae; palpifer with outer margin strongly curved at base; maxillary palp 4-segmented, with distal palpomere fusiform, longer than palpomere I–III combined, with a small, fusiform dorsal sensory area; galea (Fig. 3D3) apically large, with two acute teeth. Mandible externally rounded and weakly raised at apex; apical tooth rounded and slightly deflected ventrally; outer face with thick, white setae. Antennae with 10 antennomeres, with club elongate, slightly shorter than antennomeres 2–7 combined. **Pronotum** widest at middle; medially wider than head, with lateral margins evenly curved. Surface moderately covered with deep, setigerous punctures; lateral punctures with decumbent, scale-like, white setae. Anterior bead punctate, barely defined at middle; posterior bead complete and well defined. Anterior angles acute; posterior angles obtuse. **Scutellar shield** small, as wide as long, with posterior margin acute; basal surface with sparse, deep, setigerous punctures bearing thick, decumbent, white setae. **Elytra** shallowly rugopunctate, with three barely defined longitudinal costae. Lateral margins glabrous. **Pygidium** subtriangular and strongly concave posteriorly; whole surface moderately covered with deep, transversely fusiform punctures bearing white, transversely decumbent scale-like setae; posteromedial region with a few thin, erect setae. **Thoracic venter**: prosternum, meso- and metaventrite, and metacoxae densely covered with decumbent, scale-like, white setae; posterior prosternal process triangular, with apex acute. Metaventrite with postcoxal line medially effaced, with a row of thinner, hair-like setae. **Legs**: protibia slender, slightly wider at apex, with three small, acute outer teeth; proximal tooth reduced and largely separated from medial and distal teeth; protarsomeres I–V (Figs 1A, B, 2A, B, 3C) dorsoventrally flattened and densely setose internally; protarsomere I small, slightly surpassing the apex of protibia; II–IV somewhat caliciform and as wide as long; V as long as protarsomeres III and IV combined. Protarsal claws small, shorter than protarsomere V; anterior protarsal claw slender and laterally flattened, with a horizontal, apical cleft; posterior claw simple and shorter than anterior claw. Meso- and metafemur with outer margin coarsely crenulated; each crenulation bearing a short, thin seta. Meso- and metatibia slightly constricted near apex, with outer margin bearing one short, spine-like processes adjacent to each transverse carina (Fig. 3A, B); surface moderately covered with thick, hair-like, white setae. Inner meso- and metatibial spur longer than respective tarsomere I. Meso- and metatarsomeres I–IV slightly flattened dorsoventrally and densely setose internally. Meso- and metatarsomere V cylindrical, sparsely setose internally, longer than respective tarsomeres II–IV combined, with a short, acute internobasal tooth. Meso- and metatarsal claws slender, in flexed position as long as respective tarsomere V; anterior claw slightly thicker, with apex narrowly cleft; posterior claw simple. Meso- and metatarsal empodium with two long, apically bifurcate setae. **Abdomen** densely covered with decumbent, scale-like, white setae. Ventrite 1 medially projected, with posterior postcoxal line posteriorly extending to posterior margin of ventrite 1 and bearing dense, hair-like setae. Ventrites 1–4 with wide laterolongitudinal carinae. Ventrite 6 with posterior margin weakly emarginate. **Aedeagus** (Figs 1D, 2C, D): parameres small, about ½ length of tectum; prolongations subparallel and completely fused together to apex, with a median fusion line barely defined. Apex evenly rounded, as wide as or slightly wider than midpoint, with outer margins slightly converging. Inner surface bearing an internomedial row of setae.

**Female** (Fig. 3A–C). Length 11–11.5 mm. Width 5.8–6 mm. Females very similar to males but with body more robust, pronotum sparsely setose, protibial outer teeth stronger, pro- and mesotarsomeres moderately setose and narrower, and posterior margin of ventrite 6 evenly rounded, not emarginate.

#### Distribution (Fig. 4).

Brazil. Bahia: Encruzilhada (new state record). Espírito Santo: Linhares, Rio Bonito, Itapemirim, Santa Leopoldina. Rio de Janeiro: Petrópolis, Boa Sorte, Bom Jardim. Minas Gerais: Mar de Espanha, Águas Vermelhas, Berizal, Itumirim, Ipatinga, Lavras. São Paulo: Itú (new state record).

**Figure 4.**
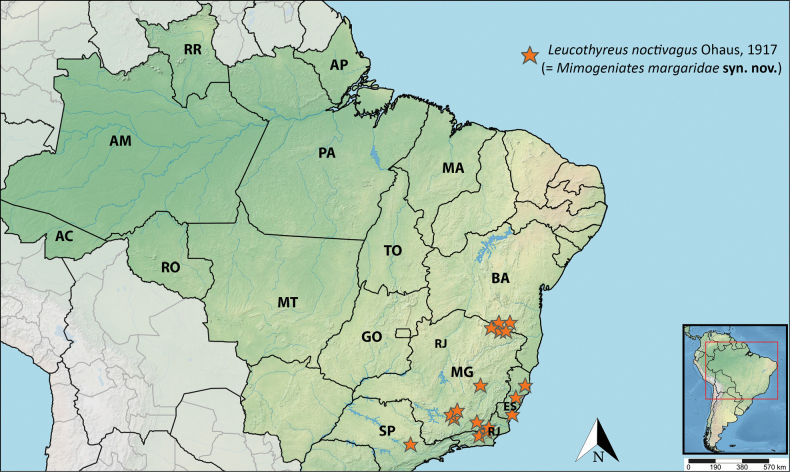
Distribution of *Leucothyreus noctivagus* Ohaus, 1917.

## ﻿Discussion

Martínez (1964) erected the monotypic genus *Mimogeniates* for his new species *M. margaridae* based on the crenulated, unprojected prementum, as well as the unprojected apical margin of labrum. Herein, based on the analysis of the type series and additional material, we synonymize this species with *Leucothyreus noctivagus* Ohaus, 1917 (new synonym).

In the original description of *L. noctivagus*, Ohaus (1917) compared this species to *L. niveicollis* Laporte, 1840. According to Jameson and Hawkins (2005), Ohaus was confused about the identity of these species and even mixed both species within an identified series of *L. niveicollis*, which is reflective of their similarity. However, *L. noctivagus* is easily distinguished from *L. niveicollis* by the lack of scale-like setae on the elytral surface (covered with scale-like setae in *L. niveicollis*) and the lack of a medioapical projection on the labium (present in *L. niveicollis*).

As revealed by a phylogenetic analysis (Bento 2024), *L. noctivagus* is nested within the *Leucothyreus* clade, and forms a subclade with the type species *L. kirbyanus*. This clade was strongly supported by 10 synapomorphies (Bremer support value = 83), including the coarsely crenulated outer margin of metafemur and the outer margin of meso- and metatibia with short, spine-like projections appeared as exclusive synapomorphies. Additional synapomorphies are: maxillary tooth III pointed, pedicel shorter than antennomere 3, scale-like setae on pronotal surface, posterior margin of pronotum beaded, and prosternal process projected between procoxae.

The close morphological similarity and phylogenetic relationship between *Leucothyreus noctivagus* and *L. kirbyanus* suggest that the median projection on the apical margins of the labrum and prementum—a diagnostic trait of the tribe Geniatini—has been lost independently multiple times during the tribe’s evolutionary history. Although uncommonly found in Geniatini, the absence of medioapical projections on the labrum and prementum, a character state based on which Martínez (1964) erected *Mimogeniates*, should not be used alone to define higher taxa. The aforementioned phylogenetic analysis provides support that this character state may have been independently derived in many unrelated lineages of geniatine scarabs (Bento 2024).

Therefore, based on the synonymy of *M. margaridae* with *L. noctivagus*, the monotypic genus *Mimogeniates* Martínez, 1964 is here synonymized within *Leucothyreus* Macleay, 1819 (new synonym). As a result of our work, the tribe Geniatini includes 12 genera and 337 species.

## Supplementary Material

XML Treatment for Leucothyreus noctivagus

## ﻿Additional information

### Use of AI

No use of AI was reported.

### Author ORCIDs

Matheus Bento https://orcid.org/0000-0001-9846-9728

Paschoal Grossi https://orcid.org/0000-0001-6601-5967

Claudio Ruy Vasconcelos da Fonseca https://orcid.org/0000-0002-1955-288X

Matthias Seidel https://orcid.org/0000-0002-4913-8778

### Data availability

All of the data that support the findings of this study are available in the main text.
